# Factors influencing the spread of pertussis in households: a prospective study, Catalonia and Navarre, Spain, 2012 to 2013 

**DOI:** 10.2807/1560-7917.ES.2016.21.45.30393

**Published:** 2016-11-10

**Authors:** Pere Godoy, Manuel García-Cenoz, Diana Toledo, Glòria Carmona, Joan A Caylà, Miquel Alsedà, Josep Àlvarez, Irene Barrabeig, Neus Camps, Pere Plans, María Company, Jesús Castilla, Maria-Rosa Sala-Farré, Carmen Muñoz-Almagro, Cristina Rius, Àngela Domínguez, for the Transmission of Pertussis in Households Working Group

**Affiliations:** 1 Agència de Salut Pública de Catalunya, Barcelona, Spain; 2 Ciber de Epidemiología y Salud Pública, CIBERESP, Madrid, Spain; 3 Institut de Recerca Biomèdica de Lleida, IRBLleida, Lleida, Spain; 4 Instituto de Salud Pública de Navarra, IdiSNA, Pamplona, Spain; 5 Universidad Pública de Navarra (UPNA), Navarre, Spain; 6 Universitat de Barcelona, Barcelona, Spain; 7 Agència de Salut Pública de Barcelona, Barcelona, Spain; 8 Hospital de Sant Joan de Dèu, Barcelona, Spain; 9 Universitat Internacional de Catalunya, Barcelona, Spain; 10 Members of the group are listed at the end of the article

**Keywords:** Pertussis, transmission, Household, vaccine, epidemiology, Chemoprophylaxis

## Abstract

We aimed to investigate transmission rates of pertussis in household contacts of cases and factors associated with transmission. A prospective epidemiological study was conducted in 2012 and 2013 to determine the incidence of pertussis among household contacts of reported cases in Catalonia and Navarre, Spain. An epidemiological survey was completed for each case and contact, who were followed for 28 days to determine the source of infection (primary case) and detect the occurrence of secondary cases. Odds ratios (ORs) were used to estimate the effectiveness of vaccination and chemoprophylaxis in preventing new cases, using the formula (1 − OR) × 100. For the 688 primary cases, a total of 2,852 contacts were recorded. The household transmission rate was 16.1% (459/2,852) and rose according to the age (> 18 years) and lack of immunisation of the primary cases, and also the age (0–18 years), family relationship (siblings and children), lack of vaccination and chemoprophylaxis of contacts. Pertussis vaccine effectiveness in preventing new cases was 65.0% (95% confidence interval (CI): 11.6 to 86.2) for full vaccination (≥ 4 doses) and 59.7% (95% CI: −6.8 to 84.8) for incomplete vaccination (< 4 doses). The effectiveness of chemoprophylaxis was 62.1% (95% CI: 40.3 to 75.9). To reduce household transmission, contacts should be investigated to detect further cases and to administer chemoprophylaxis. The current vaccination status of cases and contacts can reduce household transmission.

## Introduction

Pertussis vaccination has led to an important reduction in the incidence of the disease in children in the past 60 years [1]. However, pertussis remains a vaccine-preventable disease that causes a large number of deaths worldwide [2] and has high incidence and hospitalisation rates, even in industrialised countries [3,4].

Studies suggest that the persistence of transmission of the causative agent, *Bordetella pertussis*, is due to the fact that immunity to *B. pertussis* infection – whether acquired naturally or by vaccination – is not lifelong [5,6]. In fact, a second infection in people who have already been infected with *B. pertussis* have been reported [7]. When whole-cell vaccines (wPs) are used, protective antibodies decline by 50% over a period of 6 to 12 years [5,8]. The duration of immunity conferred by acellular vaccines (aPs) – which are used today in most industrialised countries because they are less reactogenic [9] – appears to be shorter than that conferred by wP [10,11]. Some studies suggest that aPs induce a suboptimal immune response that is unable to prevent infection, thus providing a plausible explanation for pertussis resurgence [12].

In Spain, the wP against pertussis, combined with diphtheria and tetanus toxoids (DTwP), was commercialised in the 1960s and was administered to infants (aged under 1 year) in two annual campaigns [13]. In Catalonia and Navarre, the wP was included in 1980 in the national childhood immunisation schedule, with four doses at 3, 5, 7 and 18 months of age. In 1998, the vaccination schedule was changed, reducing the age of administration and number of the wP doses at 2, 4 and 6 months of age, and included two doses of the aP (at 18 months and 4–6 years of age). In 2002, five doses of aP – diphtheria, tetanus and acellular pertussis (DTaP)/combined tetanus, diphtheria and acellular pertussis (Tdap) – were introduced into the childhood immunisation schedule, with the last dose given at the age of 4–6 years, to reduce the side effects of wP vaccination. In Spain, vaccination coverage with pertussis vaccines has been more than 90% since 1990 [13]. Nevertheless, pertussis incidence increased from less than 1 per 100,000 population in 2003 to 5.3 per 100,000 population in 2013 [13].

Studies of children worldwide hospitalised due to serious outcomes of pertussis have shown that the most frequent source of infection is in the household, due to infection by mothers or other family members (siblings, fathers, grandparents) or caregivers, who presented with symptoms of coughing that were not recognised as being due to pertussis [14-16].

Other studies of community index cases also indicate that *B. pertussis* transmission often occurs in households and that transmission rates in this setting are variable but high, depending on factors related to the pertussis cases and their contacts, such as age, sex or immune status [17,18].

The rate of secondary transmission of *B. pertussis* in Spanish households and the relative importance of family relationships and specific age groups regarding infection is unknown. Similarly, chemoprophylaxis with azithromycin is recommended for post-exposure prophylaxis [19], but its effectiveness, and that of DTwP/DTaP/Tdap vaccination, in preventing transmission in household contacts is also unknown. Such data could be valuable in the assessment of strategies to reduce the number of *B. pertussis* infections, especially in children.

The aim of our study was to investigate the sources of infection of primary cases and rates of secondary transmission of pertussis in contacts of pertussis cases in households and factors associated with transmission in Catalonia and Navarre, Spain.

## Methods

A prospective epidemiological study was conducted in 2012 and 2013 on the incidence of pertussis among household contacts of pertussis cases who were reported to the notifiable diseases systems of Catalonia and Navarre, which together have a population of 8.2 million [20].

Index cases (defined below) were reported to public health professionals from the epidemiological surveillance units of the Department of Health of Catalonia, the Public Health Agency of Barcelona and the Public Health Institute of Navarre. Each case notified was considered an index case. To be included in the study, an individual had to meet the criteria for a confirmed case (see below) and have household contacts who could be identified.

For each index case detected, an epidemiological survey of the study variables (outlined below) was completed and household contacts were identified. Each case was asked about exposure to a person with pertussis, symptoms, doses of pertussis vaccine received (registered in an official document or medical history) and preventive measures adopted (vaccination or chemoprophylaxis). As the transmission period of the disease may be as long as 21 days [21] and the incubation period in a new case seven days [22], cases and contacts were followed for 28 days to determine the source of infection (primary case), and the appearance of secondary cases.

Two samples were taken, using appropriate swabs (Dacron or Rayon for PCR) and cotton for cultures), from the posterior nasopharynx of each case and contact with pertussis-compatible symptoms for determining presence of *B. pertussis* by culture or PCR. Swabs for culture were transported in a suitable medium to ensure viability of the bacteria and swabs for PCR were resuspended in 200 µL saline solution. *B. pertussis* DNA was detected using real-time PCR amplification of the insertion sequences *Bordetella* IS481 [23]. Human RnaseP gene was used to check sample quality and detection of inhibitors of PCR reaction.

### Definitions

An index case was defined as the first reported pertussis case who generated the study of pertussis in a particular household. 

A confirmed case was defined as a person presenting clinically with a cough, together with microbiological confirmation (isolation of *B. pertussis* in culture or positive PCR test from nasopharyngeal swabs) or a person who fulfilled the clinical definition (cough for more than two weeks and at least one of the following: paroxysmal cough, inspiratory stridor, post-tussive vomiting or apnoea) and who was also epidemiologically linked to a confirmed case.

A primary case was defined as the first confirmed case of pertussis in a household to develop symptoms. 

A coprimary case was defined a confirmed case of pertussis with symptoms appearing between 0 and 6 days after those of the primary case had started. 

A secondary case was a confirmed case in whom symptoms began between 7 and 28 days after those of the primary case. 

After completion of the survey and laboratory tests, each index case and household contact was classified as a healthy contact, primary case or secondary case (confirmed microbiologically or by epidemiological link).

Household contacts were defined as all residents of the household of the primary case (cohabitants) or persons who had had contact with the primary case for more than 2 hours (to exclude sporadic contact) in the same dwelling during the transmission period of the disease (non-cohabitants) in order to detect cases among relatives and caregivers who were not household cohabitants but could have a relevant role in the epidemiological chain. We choose 2 hours to eliminate sporadic contact (with less than 2 hours of contact).

The transmission period of the disease was defined as the period of 21 days from the onset of symptoms in the primary case or five days from the onset of treatment of the primary case. 

### Study variables

Information on the following sets of variables was obtained from a face-to-face questionnaire and official records for each pertussis case and each household contact.

Demographic variables: sex, age, number of persons in a household (cohabitant or non-cohabitant) and the relationship between the household members. For contacts, the relationship with the primary case (e.g. mother, father, sibling, grandparent, child, partner, other) was recorded.

Clinical variables: date of onset of first symptom, cough lasting 2 or more weeks, number of days of persistent cough, and presence/absence of paroxysmal coughing, post-tussive vomiting, apnoea, fever, pneumonia, seizures, encephalopathy, hospitalisation.

Laboratory results: type of sample, result of culture and PCR.

Preventive measures: for study participants – all cases of pertussis (all ages) and household contacts (aged ≤ 18 years) – who had received any dose of pertussis vaccine, the number and date of administration of doses were recorded. The cut-off of 18 years was chosen because few contacts aged more than 18 years had records of their vaccinations. Vaccination status was categorised as fully vaccinated (≥ 4 doses of vaccine), undervaccinated (< 4 doses), unvaccinated (no dose), undervaccinated due to age (< 4 doses) and unvaccinated due to age (no dose). 

Chemoprophylaxis was defined as completion of antibiotic treatment (azithromycin) in a healthy contact (all ages) initiated after symptom onset of the primary case.

### Sample size

Given that the annual median number of new cases in Catalonia and Navarre was 203 [24] and the study period was 2 years, we expected to register 406 new cases during the study. Taking a mean of three household contacts (excluding the index case), we expected to register 1,218 household contacts. The median size of families in Spain is 2.5 members [25]; however, as other contacts in households, such as caregivers, were included, we decided to use a mean of three. 

The rate of transmission in households, assuming an expected level of 10% [21], was estimated to a precision of ± 1.7%. 

### Data analysis

Primary cases and contacts were described using percentages with their 95% confidence intervals (CIs) for qualitative variables, and means and standard deviation (SD) for quantitative variables.

The rate of transmission with its 95% CI was calculated using the formula:

Secondary transmission rate = (Cases detected among the household contacts of primary cases/Total number of household contacts) × 100

Primary cases were not included in the numerator or the denominator.

The risk of transmission was studied according to the characteristics of primary cases and their household contacts using the chi-squared test for qualitative variables and the ANOVA or Kruskall tests for quantitative variables, with a level of significance of p < 0.05. The strength of an association was calculated using odds ratios (ORs) and their 95% CIs.

The vaccine effectiveness (only in household contacts aged 18 years or under) and chemoprophylaxis (in all household contacts) was studied using the formula: Effectiveness = (1 − OR) × 100. The estimated ORs were adjusted using an unconditional logistic regression model produced by eliminating variables using stepwise regression in which predictive variables were carried out by the automatic backward method starting with all candidate variables and eliminating variables from p < 0.2. 

The variables evaluated in the models were vaccination status, use of chemoprophylaxis, age, sex and family relationship of the contacts, in addition to the sex, age and vaccination status of the primary case.

### Ethical aspects

The study was approved by the Ethics Committee of the Hospital Sant Joan de Deu (code: PIC-79–11). All contacts and family members were informed about the study and gave their consent to participate.

## Results

We studied 688 index cases, of whom 76.2% (524/688) were the primary cases in the household. The remainder (164/688) were secondary cases (household contacts). Thus the 688 primary cases studied (the first cases who became symptomatic in a household) comprised 524 index cases and 164 household contacts who were identified as primary cases once the study of the household was complete. 

Of these 688 confirmed primary cases, 52.8% were female, 21.9% were aged under 1 year, 42.6% 1–10 years, 14.2% 11–18 years and 21.2% more than 18 years. Primary cases had the following symptoms: cough lasting more than 2 weeks (93.6%), paroxysmal cough (84.4%), post-tussive vomiting (40.1%), inspiratory stridor (37.6%), apnoea (21.9%) and fever (10.8%) (Table 1). The frequency of symptoms experienced by primary cases aged more than 18 years was slightly different: cough lasting more than 2 weeks (98.6%), paroxysmal cough (84.4%), post-tussive vomiting (23.1%), inspiratory stridor (30.6%), apnoea (20.4%) and fever (6.8%). Of the 688 primary cases, 15.3% were hospitalised, including 63.6% (96/151) of those aged under 1 year.

**Table 1:** Characteristics of primary cases of pertussis with household contacts, Catalonia and Navarre, Spain, 2012–13 (n = 688)

Characteristic of primary case	Number	%
Sex		
Male	325	47.2
Female	363	52.8
Age in years		
< 1	151	21.9
1	24	3.5
2–3	44	6.4
4–6	76	11.0
7–10	149	21.7
11–18	98	14.2
19–40	76	11.0
> 40	70	10.2
Clinical symptoms		
Cough lasting > 2 weeks	644	93.6
Paroxysmal cough	581	84.4
Post-tussive vomiting	276	40.1
Inspiratory stridor	259	37.6
Apnoea	151	21.9
Fever	74	10.8
Laboratory confirmation (PCR and/or culture)		
Yes	504	73.3
No	184	26.7
Hospitalisation		
Yes	105	15.3
No	583	84.7
Vaccination status^a^		
Fully vaccinated	331	48.1
Undervaccinated due to age	90	13.1
Undervaccinated	15	2.2
Unvaccinated	61	8.9
Unvaccinated due to age	66	9.6
Unknown/no answer	125	18.2

^a^ Vaccination status was categorised as fully vaccinated (≥ 4 doses of vaccine), undervaccinated (< 4 doses), unvaccinated (no dose), undervaccinated due to age (< 4 doses) and unvaccinated due to age (no dose).

Laboratory confirmation (PCR and/or culture) was obtained for 73.3% (n = 504) of the primary cases and by epidemiological link in 26.7% (n = 184); 48.1% of cases were fully vaccinated (they had received ≥ 4 doses of vaccine), 13.1% were undervaccinated due to age, 2.2% were simply undervaccinated, 8.9% had received no vaccine dose and 9.6% were unvaccinated due to age (Table 1).

A total of 2,852 household contacts of the 688 primary cases were recorded, of whom 52.8% were female, 66.0% were older than 18 years, 7.3% were aged 11–18 years and 26.6% were under 11 years. About 71% of the contacts were cohabitants, i.e. they lived in the same household as the primary case. The most common family relationships among the contacts were being a mother (19.5%), father (17.9%) or sibling (18.2%) of the primary case. Some 64% of contacts aged ≤ 18 years were fully vaccinated, 13% were undervaccinated and 11% were unvaccinated (Table 2).

**Table 2:** Characteristics of household contacts of primary cases of pertussis, Catalonia and Navarre, Spain, 2012–13 (n = 2,852)

Characteristic of household contact	Number	%
Sex		
Male	1,340	47.0
Female	1,512	53.0
Age in years		
< 1	150	5.3
1	58	2.0
2–3	132	4.6
4–6	200	7.0
7–10	221	7.7
11–18	209	7.3
19–40	967	33.9
> 40	915	32.1
Household contacts		
Cohabitant	2,034	71.3
Non-cohabitant	818	28.7
Relationship to primary case		
Mother	556	19.5
Father	510	17.9
Sibling	518	18.2
Grandparent	330	11.6
Child	139	4.9
Partner	100	3.5
Other^a^	699	24.5
Number of contacts in the household		
≤ 2	226	7.9
3–4	1,133	29.7
> 4	1,493	52.3
Vaccination status (≤ 18 years)^b^		
Fully vaccinated	581	64.4
Undervaccinated due to age	94	10.4
Undervaccinated	27	3.0
Unvaccinated	49	5.4
Unvaccinated due to age	53	5.9
Unknown/no answer	97	10.8
Received chemoprophylaxis^c^		
Yes	2,284	80.1
No	406	14.2
Unknown	162	5.7

^a^ Caregiver, family friend or neighbour.

^b^ Vaccination status was categorised as fully vaccinated (≥ 4 doses of vaccine), undervaccinated (< 4 doses), unvaccinated (no dose), undervaccinated due to age (< 4 doses) and unvaccinated due to age (no dose).

^C^ Azithromycin was used.

The household transmission rate (incidence of pertussis among household contacts) was 16.1% (459/2,852) and was slightly higher when the primary case was male (16.2%), but this difference was not statistically significant. Compared with data from primary cases aged under 1 year, the household transmission rate was higher when the primary case was aged 4–6 years (OR: 1.7; 95% CI: 1.1 to 2.6), 7–10 years (OR: 1.8; 95% CI: 1.3 to 2.6), 11–18 years (OR: 1.8; 95% CI: 1.2 to 2.6), 19–40 years (OR: 4.6; 95% CI: 3.2 to 6.6) and older than 40 years (OR = 4.3; 95% CI: 3.0 to 6.3). It was also higher when the primary case was undervaccinated, unvaccinated or of unknown vaccination status (OR: 1.3; 95% CI: 1.1 to 1.6), when compared with primary cases who were fully vaccinated (Table 3). 

**Table 3:** Incidence of pertussis in household contacts by characteristic of primary cases (n = 2,852)

Characteristic of primary case	Incidence of pertussis among contacts		Odds ratio	95% CI
	%	n/total		
Sex				
Female	16.0	245/1,528	1.0	0.8 to 1.2
Male	16.2	214/1,324	Reference	
Age in years				
< 1	8.9	60/671	Reference	
1	9.6	10/104	1.1	0.5 to 2.2
2–3	10.9	25/229	1.2	0.7 to 2.0
4–6	14.4	47/326	1.7	1.1 to 2.6
7–10	15.3	91/595	1.8	1.3 to 2.6
11–18	14.9	54/363	1.8	1.2 to 2.6
19–40	31.0	90/290	4.6	3.2 to 6.6
> 40	29.9	82/274	4.3	3.0 to 6.3
Microbiological confirmation (PCR and/or culture)				
Yes	10.7	219/2,055	Reference	
No	27.0	61/226	3.1	2.2 to 4.3
Unknown	30.8	158/513	3.7	2.9 to 4.7
Hospitalisation				
Yes	10.0	48/479	0.6	0.4 to 0.8
No	17.4	403/2,312	Reference	
Number of contacts				
≤ 2	19.5	44/226	Reference	
3–4	16.0	181/1,133	0.8	0.6 to 1.1
> 4	15.7	234/1,493	0.8	0.6 to 1.1
Vaccination status^a^				
Fully vaccinated	14.1	188/1,331	Reference	
Undervaccinated/ Unvaccinated/Unknown	17.8	271/1,521	1.3	1.1 to 1.6

CI: confidence interval.

^a^ Vaccination status was categorised as fully vaccinated (≥ 4 doses of vaccine), undervaccinated (< 4 doses) or unvaccinated (no dose).

There was no statistically significant difference between the transmission rate in households with 2 or fewer contacts (19.5%), 3–4 contacts (16.0%) or more than 4 contacts (15.7%) (p > 0.05) (Table 3).

When looking at the transmission rate assessed according to variables of household contacts, the rate was slightly higher in male contacts (16.3%) than in female (15.9%), but this difference was not statistically significant. The rate was considerably higher in contacts aged under 1 year (OR: 24.6; 95% CI: 16.2 to 37.4), 1 year (OR: 8.8; 95% CI: 5.0 to 15.6), 2–3 years (OR: 3.6; 95% CI: 2.3 to 5.7), 4–6 years (OR: 3.0; 95% CI: 2.0 to 4.5), 7–10 years (OR: 2.6; 95% CI: 1.7 to 3.9) and 11–18 years (OR = 2.6; 95% CI: 1.7–3.9), compared with those aged more than 40 years (Table 4). 

**Table 4:** Incidence of pertussis in household contacts by characteristic, Catalonia and Navarre, Spain, 2012–13 (n = 2,852)

Characteristic of household contact	**Incidence of pertussis ** **among contacts**		Odds ratio	95% CI
	%	n/total		
Sex				
Female	15.9	241/1,512	1.0	0.8 to 1.2
Male	16.3	218/1,340	Reference	
Age in years				
< 1	69.3	104/150	24.6	16.2 to 37.4
1	44.8	26/58	8.8	5.0 to 15.6
2–3	25.0	33/132	3.6	2.3 to 5.7
4–6	21.5	43/200	3.0	2.0 to 4.5
7–10	19.5	43/221	2.6	1.7 to 3.9
11–18	19.1	40/209	2.6	1.7 to 3.9
19–40	9.6	93/967	1.2	0.8 to 1.6
> 40	8.4	77/915	Reference	
Household contacts				
Cohabitants	16.5	336/2034	1.1	0.9 to 1.4
Non-cohabitants	15.0	123/818	Reference	
Relationship with primary case				
Mother	8.3	46/556	1.8	1.0 to 3.4
Father	8.8	45/510	2.0	1.1 to 3.7
Sibling	25.7	133/518	7.2	4.2 to 12.6
Grandparent	4.5	15/330	Reference	
Child	61.2	85/139	33.0	17.7 to 61.5
Partner	16.0	16/100	4.0	1.9 to 8.4
Other^a^	17.0	119/699	4.3	2.5 to 7.5
Vaccination status^b^ (≤ 18 years)				
Fully vaccinated	23.8	138/581	0.11	0.07 to 0.17
Undervaccinated	52.9	64/121	0.38	0.22 to 0.68
Unvaccinated	74.5	76/102	Reference	
Received chemoprophylaxis^c^				
Yes	9.9	226/2,284	0.47	0.35 to 0.62
No	19.0	77/406	Reference	

CI: confidence interval.

^a^ Caregiver, family friend or neighbour.

^b^ Vaccination status was categorised as fully vaccinated (≥ 4 doses of vaccine), undervaccinated (< 4 doses) or unvaccinated (no dose).

^c^ Azithromycin was used.

No difference in transmission rate was observed between contacts who were cohabitants and those who were non-cohabitants (with exposure for more than 2 hours in the household of the primary case). However, the transmission rate was higher in siblings (OR: 7.2; 95% CI: 4.2 to 12.6) and children (OR: 33.0; 95% CI: 17.7 to 61.5) of primary cases (Table 4). 

Vaccine effectiveness in household contact aged ≤ 18 years was 89% (95% CI: 83 to 93) in reducing transmission in contacts vaccinated with 4 or fewer doses and 62% (95% CI: 32 to 78) in undervaccinated contacts. 

Chemoprophylaxis in all contacts had an effectiveness of 53% (95% CI: 38 to 65) in avoiding new cases.

In the multivariate analysis, the effect of vaccination and chemoprophylaxis for contacts in avoiding new cases was still seen. Vaccine effectiveness in reducing transmission in contacts aged ≤ 18 years was 65.0% (95% CI: 11.6 to 86.2) for full vaccination and 59.7% (95% CI: −6.8 to 84.8%) for undervaccinated contacts. The adjusted effectiveness of chemoprophylaxis, based on adjusted ORs (Table 5), in all contacts was 62.1% (95% CI: 40.3 to 75.9).

**Table 5:** Multivariate analysis of the effectiveness of pertussis vaccination and chemoprophylaxis of household contacts in reducing household transmission, Catalonia and Navarre, Spain, 2012–13

Characteristic of household contact	Adjusted odds ratio^a^	95% CI	p value
Vaccination status^b^ (≤ 18 years)			
Fully vaccinated	0.350	0.138 to 0.884	0.026
Undervaccinated	0.403	0.152 to 1.068	0.067
Unvaccinated	Reference		–
Received chemoprophylaxis^c^			
Yes	0.379	0.241 to 0.597	0.001
No	Reference		–

CI: confidence interval.

^a^ Adjusted by age of contacts, sex of contacts, relationship with primary case, sex of primary case, age of primary case and pertussis vaccination status of primary case.

^b^ Vaccination status was categorised as fully vaccinated (≥ 4 doses of vaccine), undervaccinated (< 4 doses) or unvaccinated (no dose).

^C^ Azithromycin was used.

## Discussion

The results of this study show that the rate of household transmission of pertussis in Spain in 2012 and 2013 was high, especially in contacts aged under 18 years, siblings and children of a primary case, unvaccinated contacts and those who had not received chemoprophylaxis.

Household transmission of pertussis is known to be related to the characteristics of primary cases and their contacts [26]. We found increased transmission in households of primary cases aged 18–40 years and those older than 40 years. In the age group 18–40 years, this could be due to closer contact between children and the primary case, especially mothers, due to dependence [15,27]. For primary cases aged more than 40 years, the increased rate of transmission might be due to atypical clinical presentation, possibly resulting in important diagnostic delays and therefore more opportunities for transmission [16,27]. Lack of vaccination or undervaccination of the primary case also resulted in an increased transmission rate, as observed in other studies [28,29], showing that although full vaccination may not avoid the disease for some cases, it may reduce transmission from the primary case.

In our study, 35.4% of primary cases were adolescents (11–18 year-olds) or adults (> 18 years). Other studies also suggest that adolescents and adults are an important reservoir of the pathogen and source of transmission to children, who are more vulnerable to infection and susceptible to serious complications [28]. In a report published in 1995, Wirsing von König et al. studied pertussis cases in 122 homes in 1995 in an area of Germany with very low vaccination coverage and estimated that adults were the source of infection in 15% of cases [18]. Later, Baptista et al. studied pertussis cases in 57 homes in Recife, Brazil, in 2003 and found that adults were the primary source of infection in 21.1% of cases [21,30]. Deen et al. studied 39 homes and 255 exposed persons in Los Angeles, United States, in 1995: in 53% of households, the primary case was aged older than 12 years [31]. Sala-Farré et al. investigated 59 clusters in an area of Barcelona in 2011 and found that the most frequent primary cases were children aged 5–9 years (29%), followed by adults aged 30–39 years (22%) [32].

In Catalan children in 2001 hospitalised due to severe symptoms of pertussis [33], the source of infection was determined for 63% of cases and for 44.6% of those whose infection source was determined, the source was an adolescent or adult. It is recognised that adolescents and adults may act as a source of infection of children [14], but in these age groups the disease is often not diagnosed and is generally under-detected [34]. In a study in Massachusetts, United States, in 1981 to 1991 Marchant et al. [23,35] found an increase in the incidence of confirmed cases in adolescents aged 11–19 years from 3 per 100,000 population to 12.9 per 100,000 population, after facilitating general practitioners’ access to serological diagnoses. In another study in Catalonia in 2013, the prevalence of *B. pertussis* infection in the previous 12 months was 1.8% in women of childbearing age (15–49 years), which suggests there is potentially a high risk for newborns [36].

Studies in various countries that included children hospitalised due to severe disease have shown that the most frequent sources of infection were mothers or other family members (fathers, teenage siblings and grandparents) who presented with coughing that had not been recognised as due to pertussis [16,21,27,37].

The rate of familial transmission from primary cases has been estimated in some studies. In the 1990s, Wirsing von König et al. found a high transmission rate of 26.7% in adult household contacts in an area of Germany with very low vaccination coverage [18] and in 2003, in Brazil, Baptista et al. found a rate of secondary transmission of 12.6% in adult household contacts [30].

In our study, we observed no differences between the number of contacts and transmission rate in the household. Similarly, in the study of Wirsing von König et al. the overall attack rate in adult contacts was independent of the family size [18] but an ecological study from 2009–13 in Minnesota, United States, reported a greater rate of pertussis in counties with a larger average household size [38].

The main characteristics of contacts with an increased transmission rate in our study were being 0–18 years of age, the sibling or child of a primary case, not vaccinated or undervaccinated and not receiving chemoprophylaxis. In terms of age, we observed a reduction of transmission in the 11–18-year age group and in adults compared with that of the other age groups. This may be due to vaccination with wP, as suggested by the World Health Organization (WHO) position paper on pertussis vaccines [9]. Reduced transmission in household adults was observed in the study of Baptista et al. in Recife, Brazil, in 2003. Some 87% of adults exposed to pertussis in the household did not acquire the disease: this was attributed to naturally acquired immunity [30]. In Catalonia and Navarre, five doses of aP were introduced into the official vaccination schedule in 2002 and therefore it may be assumed that most children aged under 11 years in our study were vaccinated with the aP. Specific responses to these changes, such as an adolescent booster dose (after the dose given at age 4–6 years) and additional booster doses in adults, may be required. 

Pertussis has re-emerged as an important public health concern in Europe since the current aP replaced the older wP. Warfel et al. showed that non-human primates receiving aP were protected from severe symptoms but not infection, and readily transmitted *B. pertussis* to contacts [39]. Key differences in T-cell memory suggest that aP vaccination induces a suboptimal immune response that is unable to prevent infection and provide a plausible explanation for pertussis resurgence [39]. Various studies suggest that attaining herd immunity will require the development of improved vaccination strategies that prevent *B. pertussis* colonisation and transmission [34,39,40]. 

The increased risk of transmission to siblings of primary cases seen in our study has also been observed by others [27,41]. The adjusted vaccine effectiveness of 65% in avoiding new cases in household contacts aged ≤ 18 years is similar to or higher than that observed in other studies [42]. Sheridan et al. found an effectiveness of 53% or 64%, depending on the method of calculation used [43]. However, a position paper by the World Health Organization (WHO) [9] and a systematic review published in the Cochrane database [44] suggest the effectiveness is somewhat higher: 84–85% in preventing typical whooping cough and 71–78% in preventing mild pertussis disease. The effectiveness of chemoprophylaxis with azithromycin in our study in preventing transmission was high (62.1%), suggesting that the detection of pertussis cases, analysing their contacts, and chemoprophylaxis may reduce household transmission, as has been suggested by others [21]. The evidence for the effectiveness of chemoprophylaxis in reducing transmission in household contacts is weak and based on expert opinion [45,46]. The results of our study and a recent cost–utility analysis [47] support the use of chemoprophylaxis in household contacts.

Our study has some limitations. First, it was based on notified cases of pertussis, which are known to be underdetected [35]. However, on the basis of the selection of study cases (confirmed cases with household contacts), an active search for contacts with pertussis symptoms was carried out using the survey and the taking of samples from all symptomatic household contacts of primary cases. To ensure all cases were detected, contacts were followed for 28 days from confirmation of the index case. Nevertheless, there may have been transmission due to asymptomatic cases beyond the 28 days of follow-up and the incidence of pertussis may be underestimated. We may not have identified individuals in a household who had recently been infected but may not have reported any specific symptoms. Thus, what is measured and presented in this study is the effectiveness of preventing clinically notifiable disease and not the prevention of infection. Second, vaccination status was collected by documented evidence of vaccination in an official document or medical records: some patients could have been classified as unvaccinated due to vaccination not being recorded, but if such a mistake applies equally to household contacts who remain healthy and those who become pertussis cases, it should not alter the estimated vaccine effectiveness. Third, chemoprophylaxis was recommended to all contacts without symptoms after detection of the index case. Some contacts who received chemoprophylaxis might appear as cases due to continuous exposure to other cases of pertussis in the household and, therefore, the effectiveness of chemoprophylaxis may be underestimated. However, our estimate was obtained after having followed routine pertussis control practices and may be a good estimate of the expected effectiveness when chemoprophylaxis is prescribed by public health services.

In conclusion, the results of our study suggest that in order to reduce household transmission household contacts should be investigated to detect secondary cases and administer chemoprophylaxis rapidly. All contacts who have not received the correct number of doses of pertussis vaccine according to the vaccination schedule should be vaccinated, in addition to receiving chemoprophylaxis. The incidence rate was lower in fully vaccinated individuals and therefore cases could be avoided in the future, although not the immediate future, as pertussis vaccine is not effective as post-exposure prophylaxis [47]. The previous pertussis vaccination status of cases and contacts is important in reducing the rate of household transmission. The administration of an additional dose of vaccine in adolescents and adults (especially those in contact with children) could also help to reduce the transmission rate [42]. Nevertheless, there is now increasing evidence that protection following booster doses of aP vaccines wanes faster in individuals primed with aP rather than with wP vaccines [9,34,39,40]. Such vaccination programmes have an impact in directly targeted populations, but there is as yet no substantial evidence that they have had an important impact on severe pertussis in infants. Thus, WHO recommends that national programmes consider vaccinating pregnant women with one dose of Tdap (in the second or third trimester and preferably at least 15 days before the end of the pregnancy) in addition to routine primary infant pertussis vaccination [9]. Ongoing surveillance of pertussis will be critical to monitor the changing epidemiology as the first ‘all-aP’-primed cohorts reach adulthood.

## Notes

**Transmission of Pertussis in Households Working Group:** The members of the Transmission of Pertussis in Households Working Group (PI11/02557) are as follows: Miquel Alsedà (MA), Josep Alvarez (JA), Cesar Arias (CA), Irene Barrabeig (IB), Neus Camps (NC), Glòria Carmona (GC), Mónica Carol (MCar), Maria Company (MC), Joaquim Ferràs (JF), Glòria Ferrús (GF), Mireia Jané (MJ), Sofia Minguell (SM), Raquel Rodríguez (RR), María-Rosa Sala (M-RS), Roser Torra (RT) (Agència de Salut Pública de Catalunya), Pere Godoy (PG), Pedro Plans (PP) (Agència de Salut Pública de Catalunya and CIBERESP), Inma Crespo (IC), Diana Toledo (DT), Àngela Domínguez (AD) and Rubén Solano (RS) (Universitat de Barcelona and CIBERESP), Joan Caylà (JCay), Sara Lafuente (SL) and Cristina Rius (CR) (Agència de Salut Pública de Barcelona and CIBERSP), Manuel García-Cenoz (MG-C), Rosana Burgui (RB), Jesús Castilla (JCast) (Instituto de Salud Pública de Navarra, Pamplona and CIBERESP), Ana Valero-Rello (AV-R), Iolanda Jordan (IJ) and Carmen Muñoz-Almagro CM-A) (Hospital de Sant Joan de Dèu, Barcelona).
